# Supplementary material for: Structurally robust lithium-rich layered oxides for high-energy and long-lasting cathodes
Source: Nat Commun. 2024 Feb 12;15:1288. doi: 10.1038/s41467-024-45490-x (PMC10861561; doi:10.1038/s41467-024-45490-x)
Supplement: Supplementary file 1 — Supplementary Information [file 41467_2024_45490_MOESM1_ESM.pdf]

Supplementary File

Structurally robust lithium-rich layered oxides for high-energy and long-lasting cathodes

Ho-Young Jang[1, †], Donggun Eum[1, 2, †, ‡], Jiung Cho[3], Jun Lim[4], Yeji Lee[4], Jun-Hyuk Song[1], Hyeokjun Park[5], Byunghoon Kim[1, 2], Do-Hoon Kim[1], Sung-Pyo Cho[6], Sugeun Jo[7], Jae Hoon Heo[1], Sunyoung Lee[1], Jongwoo Lim[7] and Kisuk Kang[1, 2, 8, 9]*

[1]Department of Materials Science and Engineering, Institute for Rechargeable Battery Innovations, Research Institute of Advanced Materials, Seoul National University, 1 Gwanak-ro, Gwanak-gu, Seoul 08826, Republic of Korea.

[2]Center for Nanoparticle Research, Institute for Basic Science (IBS), Seoul National University, 1 Gwanak-ro, Gwanak-gu, Seoul 08826, Republic of Korea

[3]Seoul Western Center, Korea Basic Science Institute (KBSI), 150 Bugahyeon-ro, Seodaemun-gu, Seoul, 03759, Republic of Korea

[4]Pohang Light Source-II, Pohang University of Science and Technology (POSTECH), 80 Jigok-ro 127 beon-gil, Nam-gu, Pohang 36763, Republic of Korea

[5]Interdisciplinary Materials Measurement Institute, Korea Research Institute of Standards and Science (KRISS), Daejeon 34113, Republic of Korea

[6]National Center for Inter-University Research Facilities, Seoul National University, 1 Gwanak-ro, Gwanak-gu, Seoul 08826, Republic of Korea

[7]Department of Chemistry, College of Science, Seoul National University, 1 Gwanak-ro, Gwanak-gu, Seoul 08826, Republic of Korea

[8]Institute of Engineering Research, College of Engineering, Seoul National University, Seoul 08826, Republic of Korea

[9]School of Chemical and Biological Engineering, College of Engineering, Seoul National University, 1 Gwanak-ro, Gwanak-gu, Seoul 08826, Republic of Korea

[†]These authors contributed equally to this work.

[‡]Present address: Department of Materials Science and Engineering, Stanford University, Stanford, CA, USA.

[*]Corresponding author: Kisuk Kang (E-mail: matlgen1@snu.ac.kr)

This supplementary information file includes:

Notes S1–S4

Figures S1–S27

Tables S1-S2

References 1−9

Supplementary Notes

Supplementary Note 1. In order to carefully select our experimental set, we established ternary phase diagram for the P2-type sodium layered phases (see Supplementary Fig. S1). $Na_{5/6}MO_2$ (M=Ni, Mn) and $Na_{5/6}(Li_{5/16}Mn_{13/18})O_2$ were selected for the end members which are analogous to $LiMO_2$ and $Li(Li_{1/3}Mn_{2/3})O_2$ (M=Ni, Mn) in the conventional O3-type LRLOs phase diagram[1], in order to preserve the general chemical formula and the oxidation state of Mn as 4+, respectively. The five compositions were then decided on the $Na_{5/6}Ni_{1/2}Mn_{1/2}O_2$-$Na_{5/6}(Li_{5/16}Mn_{13/18})O_2$ tie line to contain varied Li contents from 0.15 to 0.25 with 0.025 increment. This enables to maintain the TM oxidation states but control the Li-richness throughout the materials, thereby effectively regulating the amount of anionic redox after Na/Li ion-exchange process (Methods) as verified in Supplementary Figs. S9-11 and Table S1.

Supplementary Note 2. STXM analysis is widely accepted as a reliable method for exploring the bulk redox states of the oxygen-redox-based materials[1,2]. The new peaks at 528.6 and 531.2 eV in the differential absorbance spectrum account for hole creation in the TM_{3d}–O_{2p} hybridized bands and the lone-pair O_{2p} states, respectively[1-3], which are related to the activity of cationic and anionic redox reactions during charging, respectively. Accordingly, two regions (Region I: 527.8–529.2 eV, Region II: 530.6–531.8 eV; shaded in blue and yellow, respectively) were distinguished based on this prior knowledge (Supplementary Fig. S11a), and the integral values of each region upon charging for all five electrodes with different Li contents in the TM layer are shown Supplementary Fig. S11b. As expected, it was demonstrated that the anionic redox of Region II becomes the dominating reaction over the cationic redox of Region I as the composition is changed from $LL_{0.15}$ to $LL_{0.25}$. It should be noted that the integrated area portion values in Supplementary Fig. S11b show a discrepancy with the redox portion values estimated in Supplementary Fig. S10, particularly for $LL_{0.225}$ and $LL_{0.25}$. This is speculated to be the result of the unwanted surface oxygen activation reaction during high-voltage cell operation[4], which is hard to detect through bulk-sensitive STXM analysis.

Supplementary Note 3. We conducted *ex situ* Raman spectroscopy analysis on the series of O2-type LRLOs at the various states of charge to investigate local structural changes in bonding character or cation ordering due to the oxygen redox (Supplementary Fig. S17). In all the electrodes, characteristic peaks of the layered structure at 372.7, 405.3, 428.4, 484.2, and 606.4 cm^{-1} were similarly present in the initial state, with each peak assigned to the F_{2g}, A_g/B_g, E_g, and A_{1g} vibrational modes (Supplementary Fig. S17a)[5,6]. Common to all electrodes, the intensity of most of these peaks tended to weaken during charging and reversibly strengthen during the subsequent discharge process. One of the observable findings is the change in the A_{1g} vibrational mode in the 520–680 cm^{-1} range (dotted box area in Supplementary Fig. S17a), which is representative of the TM–O bond stretching. We thus deconvoluted the peaks in this region for the electrodes in the initial, 4.6-V-charged, and 2.0-V-discharge states, and the results are displayed in Supplementary Fig. S17b. In the 4.6-V-charged LL_y, a new peak at 643.9 cm^{-1} ($A_{1g}^{defective}$, pink shaded region) commonly emerged, which could be attributed to the out-of-plane TM migration with a new TM coordinating environment according to previous studies[5,6]. Simultaneously during this process, the original A_{1g} mode was suppressed and shifted to a lower wavenumber. Upon discharging, such transition of A_{1g} mode was restored, once again demonstrating the reversibility of TM migration in the O2 structures. Supplementary Fig. S17c presents the peak area ratio of $A_{1g}^{defective}$ to (A_{1g}+E_g) and the degree of A_{1g} shift at each 4.6-V-charged electrode, which implies the fraction of the defective layered structures to the intact layered structures (extent of TM migration)[5] and the amount of lattice shrinkage, respectively. The calculated peak area ratio was notably escalated as the electrodes have higher anionic redox activity, from 0.678 in $LL_{0.15}$ to 1.11 in $LL_{0.25}$. At the same time, the A_{1g} mode also shifted further left as the composition is changed from $LL_{0.15}$ to $LL_{0.25}$. All of these observations infer that with more oxygen oxidation involved, a greater amount of transition metal migration is coupled at the delithiated state; however, those migrations are reversibly recovered, reflecting

the structural robustness of O2-type layered structure even with high level of anionic redox reaction.

Supplementary Note 4. To elucidate the facile cation migration in the oxygen-redox-dominant electrodes, first-principles calculations were conducted for the three representative compositions: $LL_{0.15}$, $LL_{0.2,}$ and $LL_{0.25}$ (Supplementary Fig. S21). For the $LL_{0.15}$, $LL_{0.2,}$ and $LL_{0.25}$ electrodes, the composition of the simulated model was $Li(Li_{0.17}Ni_{0.21}Mn_{0.62})O_2$, $Li(Li_{0.21}Ni_{0.12}Mn_{0.67})O_2$ and $Li(Li_{0.25}Ni_{0.04}Mn_{0.71})O_2$, respectively, as shown in Supplementary Table 2. Although there were slight differences from the experimental compositions, the simulation models are plausible supercells to obtain the TM migration energetics given that the Li content in the TM layer, which regulates oxygen redox activity, is sufficiently altered. The migration energy was calculated for each supercell in which the same amount of lithium ions in the lithium layer was removed (20 out of 24 Li extracted) to simulate the actual charging situation. Once the SOC of the electrodes is equivalent, oxygen ions are oxidized the most in the simulated $LL_{0.25}$ due to the lack of cationic redox capability, which was theoretically confirmed by estimating the charge distribution (Supplementary Fig. S22). In this case, the corresponding cation migration energies of the simulated $LL_{0.15}$, $LL_{0.2,}$ and $LL_{0.25}$ electrodes were –0.26, –0.71, and –1.23 eV, respectively. This indicates that as the Li richness in the TM layer increases, the structures become more stable when the TM migration occurs, which is highly compatible with experimental consequences. These results are also consistent with previous studies that cations inevitably migrate to other sites to stabilize the oxidized oxygen network[3,7,8]. Therefore, we identified that, as the oxygen redox becomes the predominant reaction in the entire redox reaction, the TM migration proceeds more actively with shrinking the lattice of crystal structure. Nevertheless, since there is a strong coupling between the lattice oxygen oxidation and cation migration[2,3,7], these two factors cannot be completely decoupled and would inevitably influence the lattice contraction simultaneously.

Supplementary Figures

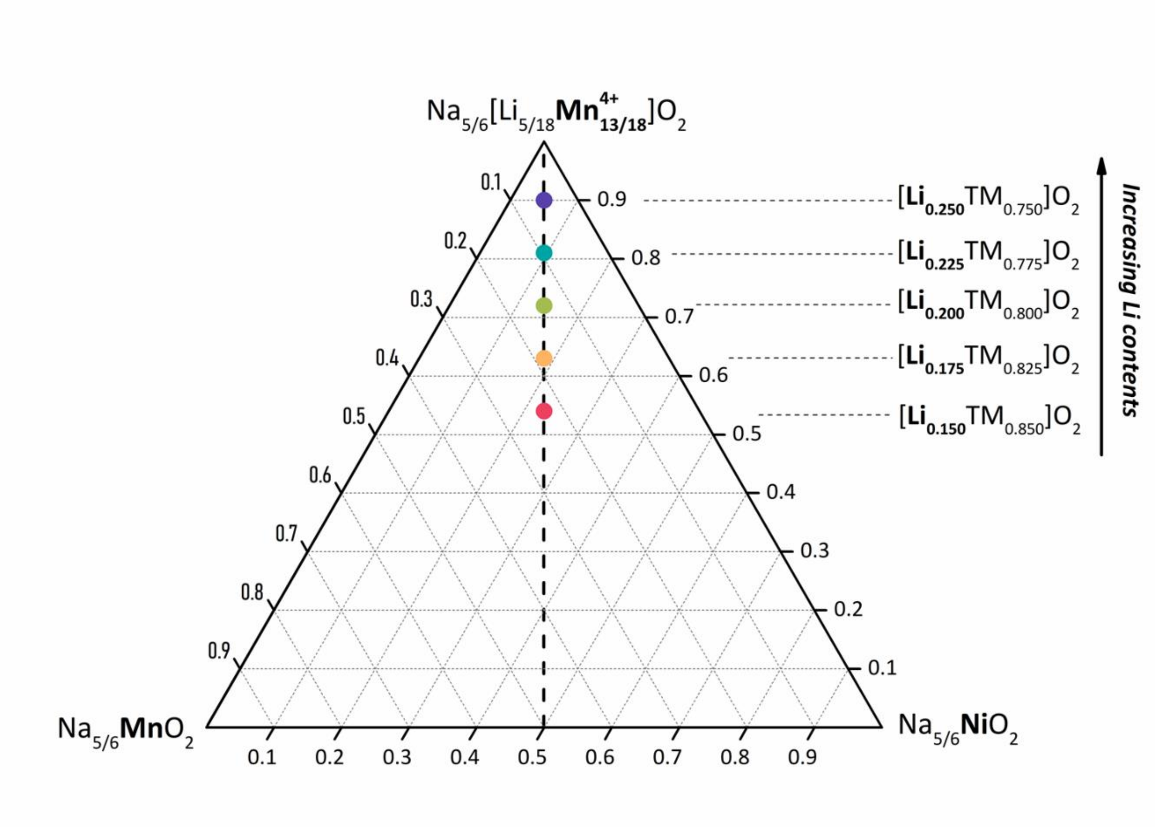

Fig. S1. Ternary phase diagram to determine five P2-type sodium layered oxides. Targeted compositions are marked on the dashed midline with the specific Li and TM ratio in TM layer assigned on the right diagram.

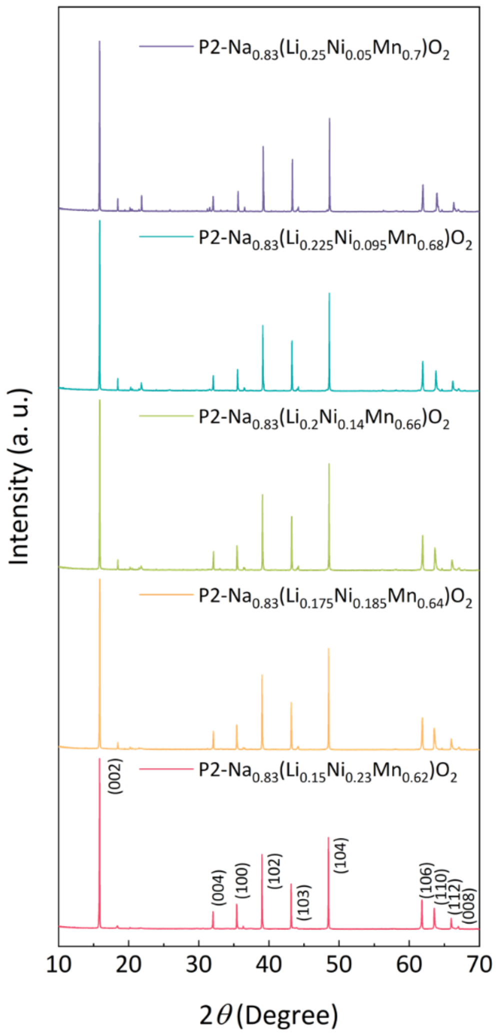

Fig. S2. HRPD patterns of P2-type sodium layered oxides determined in Fig. S1.

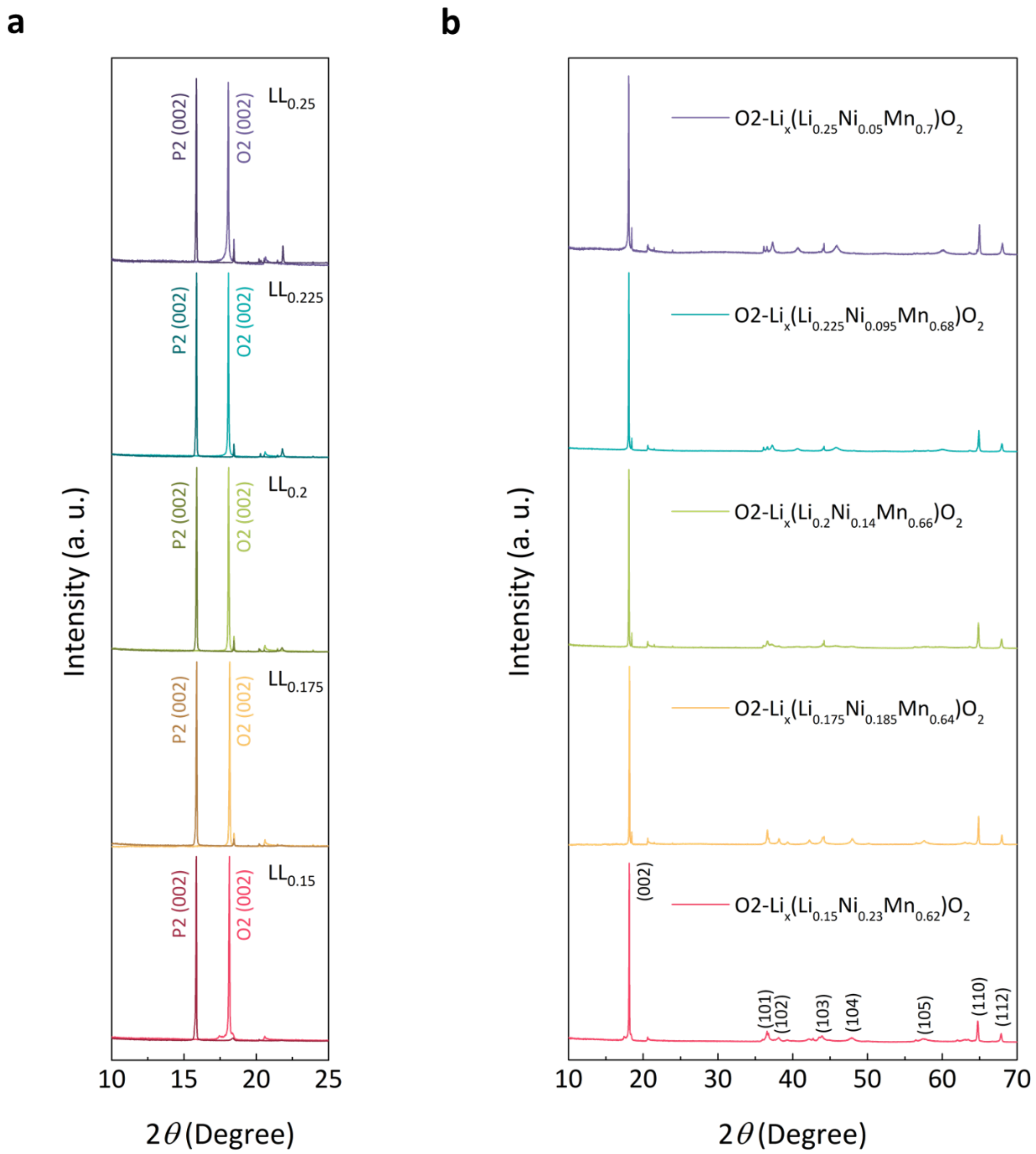

Fig. S3. **a**, Comparisons of (002) Bragg peaks between P2- and O2-type layered oxides. The peaks generally shift rightward after Na/Li ion exchange. **b**, HRPD data for O2-type LL_ys (y=0.15, 0.175, 0.2, 0.225, 0.25). Representative Bragg peaks of O2-LRLOs[5,6] are indexed.

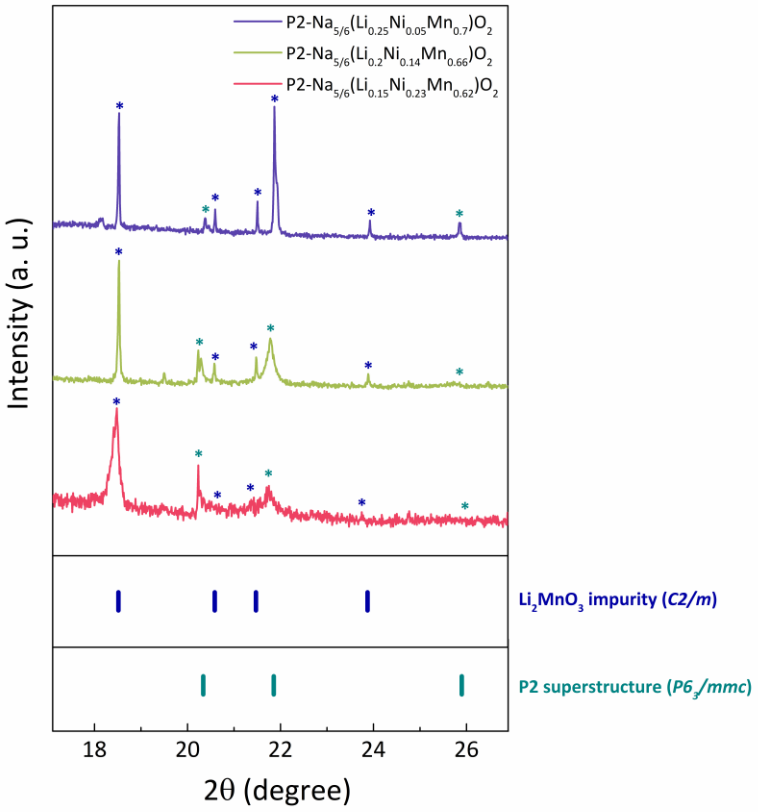

Fig. S4. HRPD patterns for P2-$LL_{0.15}$, P2-$LL_{0.2}$, and P2-$LL_{0.25}$ in 17 – 27° region and theoretical Bragg positions of O3-type Li2MnO3 impurity (navy) and superstructure patterns of P2-type sodium (cyan).

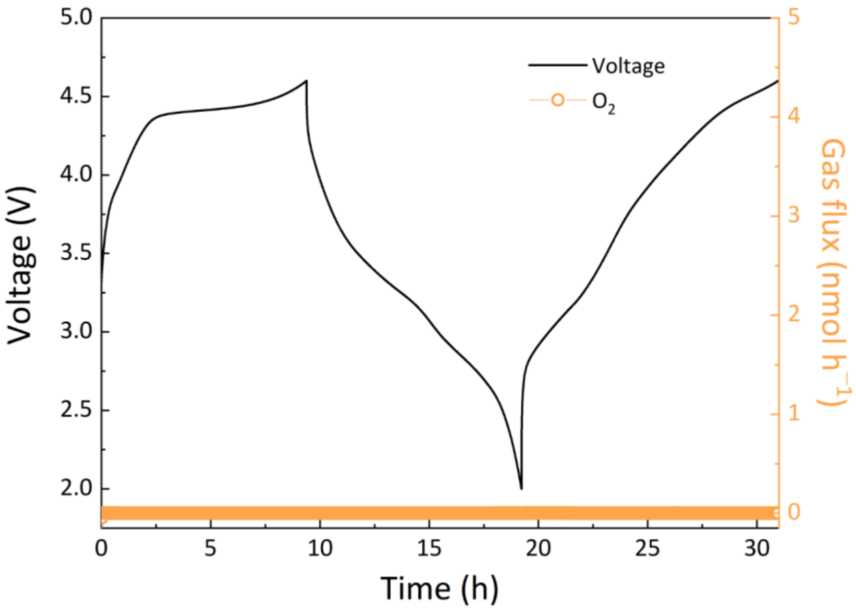

Fig. S5. Voltage profile and differential electrochemical mass spectrometry (DEMS) result of $LL_{0.25}$ for 1.5 cycles.

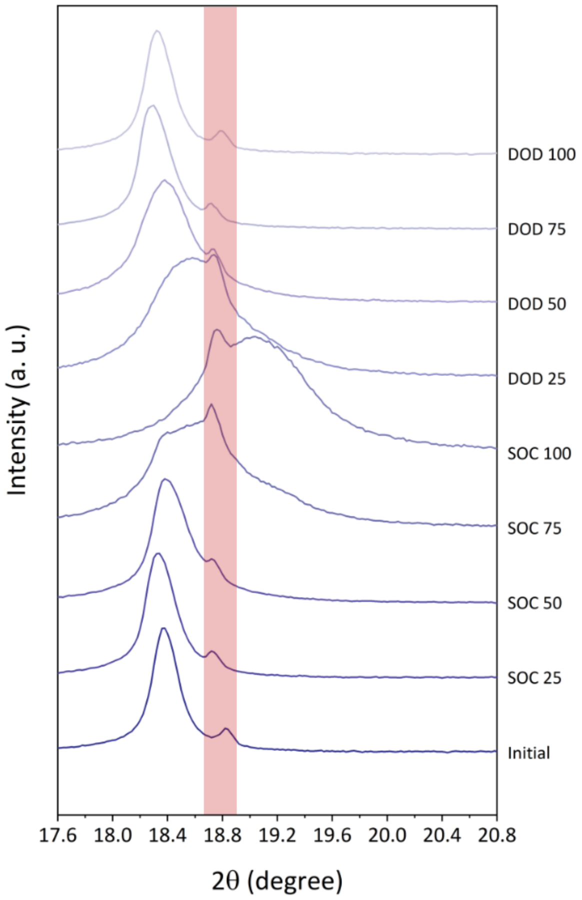

Fig. S6. *Ex-situ* XRD patterns for $LL_{0.25}$ in 2nd cycle. The red shaded region indicates the (003) peak of O3-phase.

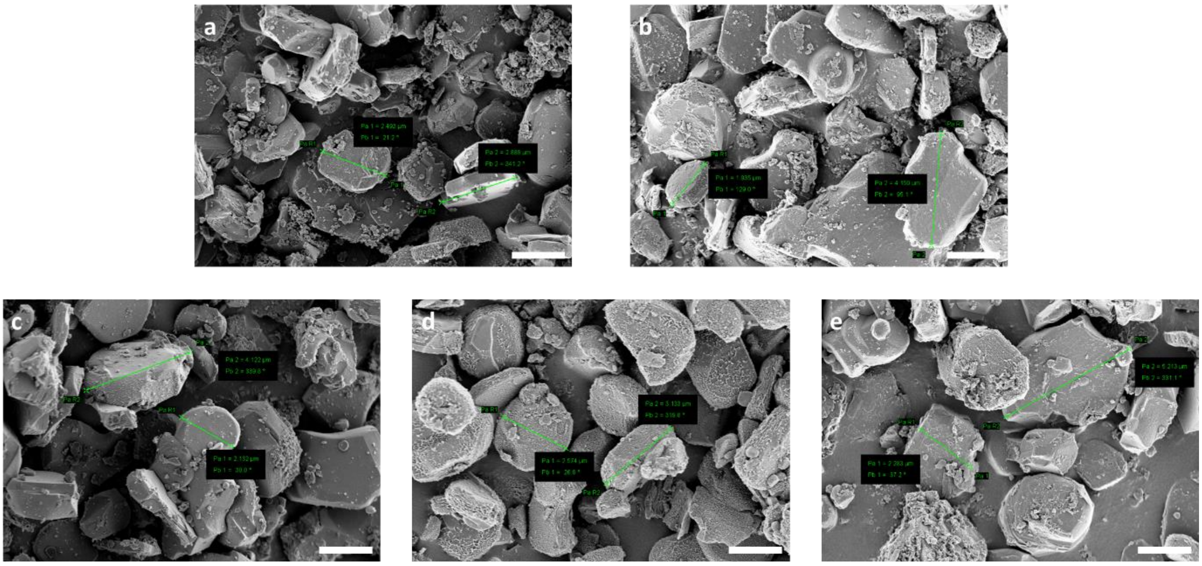

Fig. S7. Scanning electron microscopy (SEM) images of LL$_y$s (y=0.15 (**a**), 0.175 (**b**), 0.2 (**c**), 0.225 (**d**), and 0.25 (**e**)). (Scalebar: 2 μm)

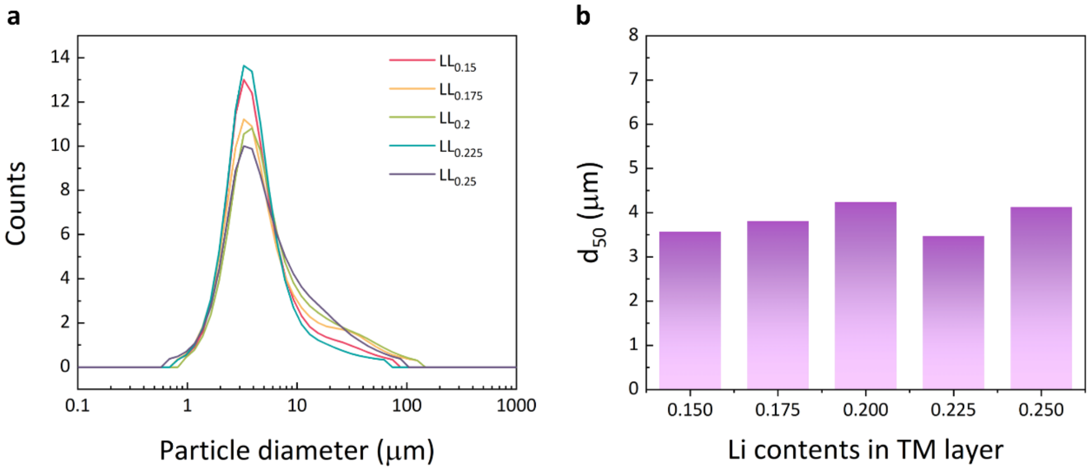

Fig. S8. **a**, PSA results and **b**, median particle size (d_{50}) of LL_ys

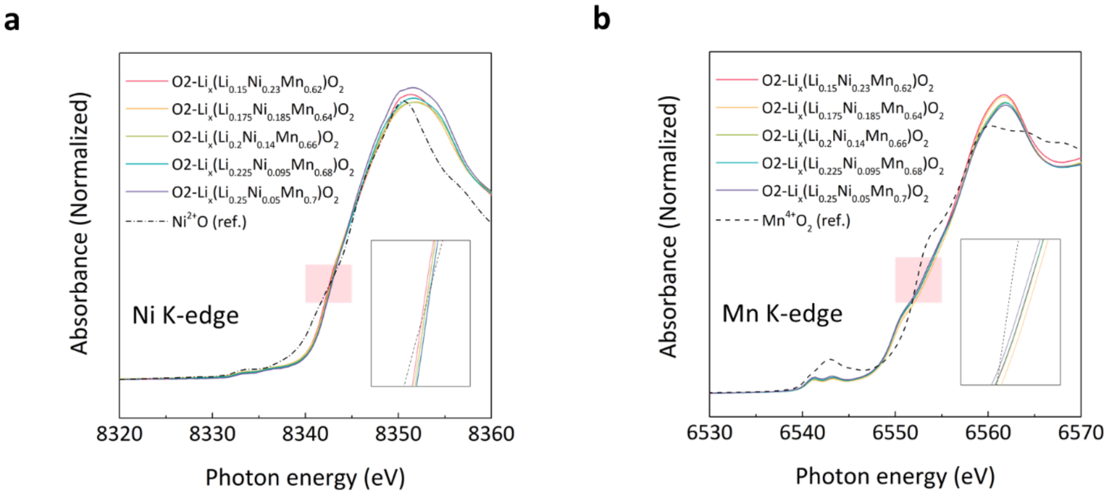

Fig. S9. XANES spectra for **a**, Ni K-edge and **b**, Mn K-edge for LL_{ys} with reference spectra. Red square areas are enlarged in insets, showing the half-normalization regions for each spectrum.

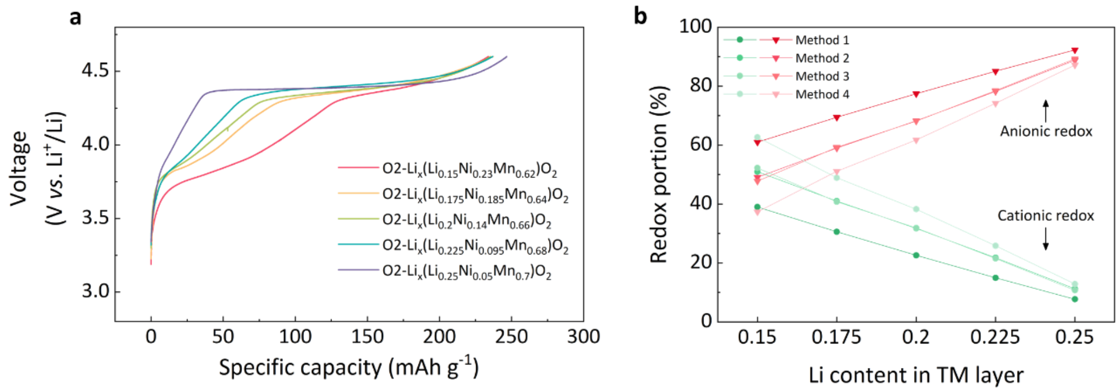

Fig. S10. **a**,1st electrochemical charge curves for O2-LRLOs. **b**, cationic / anionic redox portion calculation in various methods depending on the material composition and amount of Li utilization. To estimate the redox contribution of TM and O during the first cycle, the oxidation states for TMs were considered identically as (Mn^{4+})[2,6,9] and ($Ni^{2.33+}$) based on the nominal compositions (Supplementary Fig. S1) and XAFS results (Supplementary Fig. S9) where only Ni^{2+}/Ni^{3+}/Ni^{4+} is responsible for TM redox and rest of Li extraction is charge-compensated by O redox. Calculations with method 1 and 2 were progressed under nominal compositions, considering the full Li extraction and electrochemical Li utilization based on **a**, respectively. Method 3 and 4 follow the compositions determined by ICP results (Table S1), while the degree of Li extraction corresponds to that of method 1 and 2, respectively.

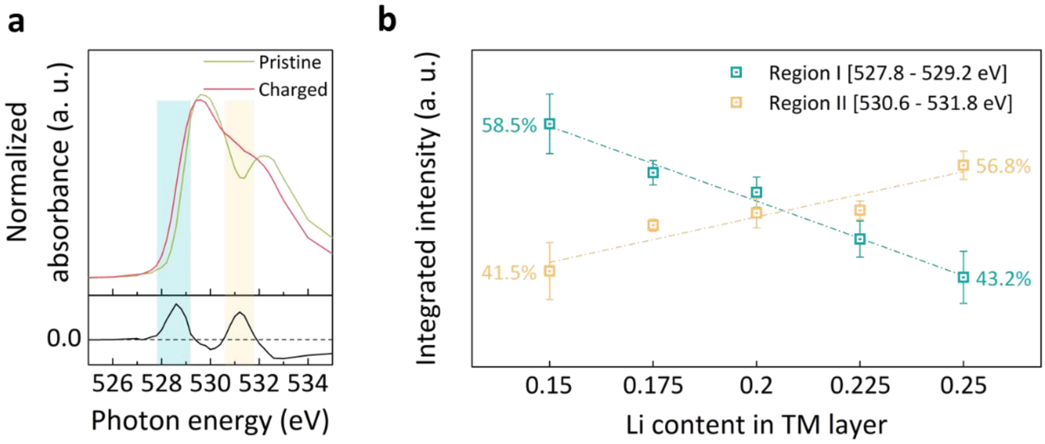

Fig. S11. a, Spatially averaged STXM differential spectra of O K-edge for the pristine and 4.6-V-charged $LL_{0.25}$, with the two positive differential regions designated as Region I (blue shaded) and Region II (yellow shaded). **b**, Integrated intensities of Region I and II for all LL_ys. Details for STXM quantification are provided in Supplementary Note 2.

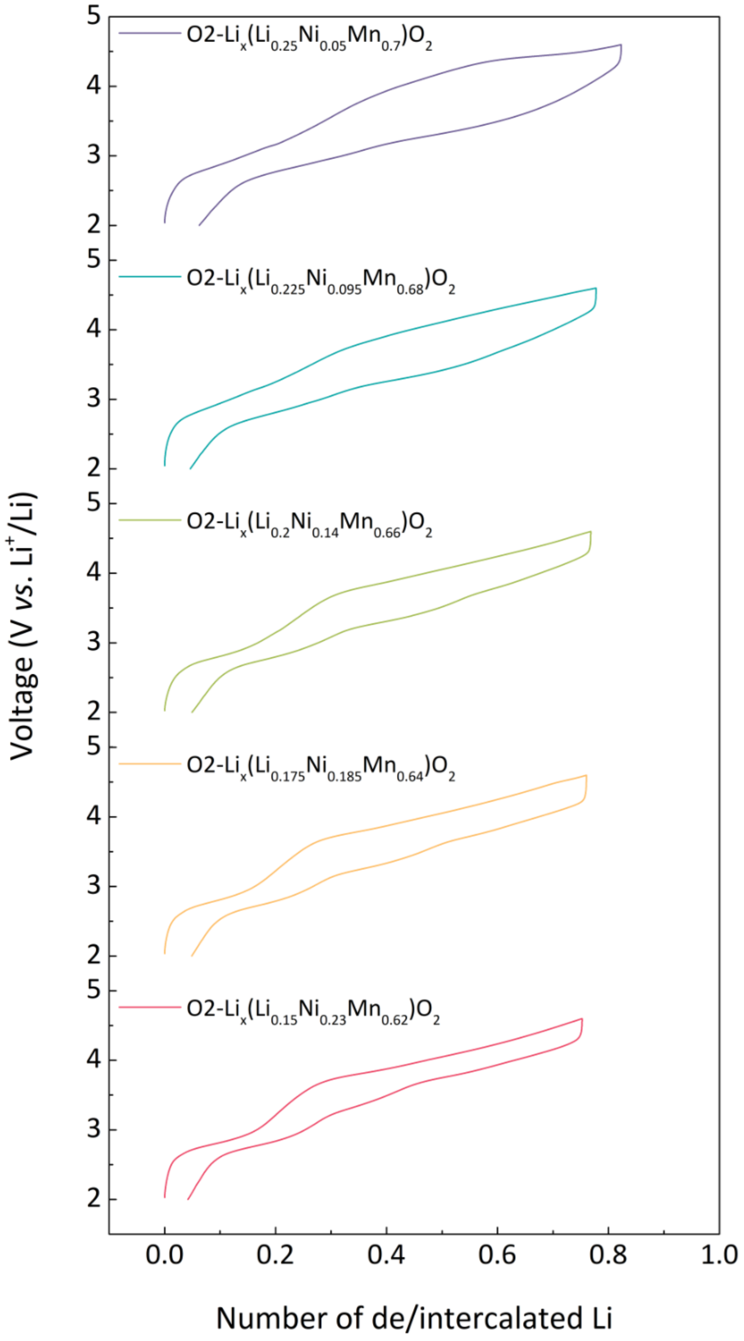

Fig. S12. Voltage profiles of LL_ys plotted against the number of (de)intercalated Li ions.

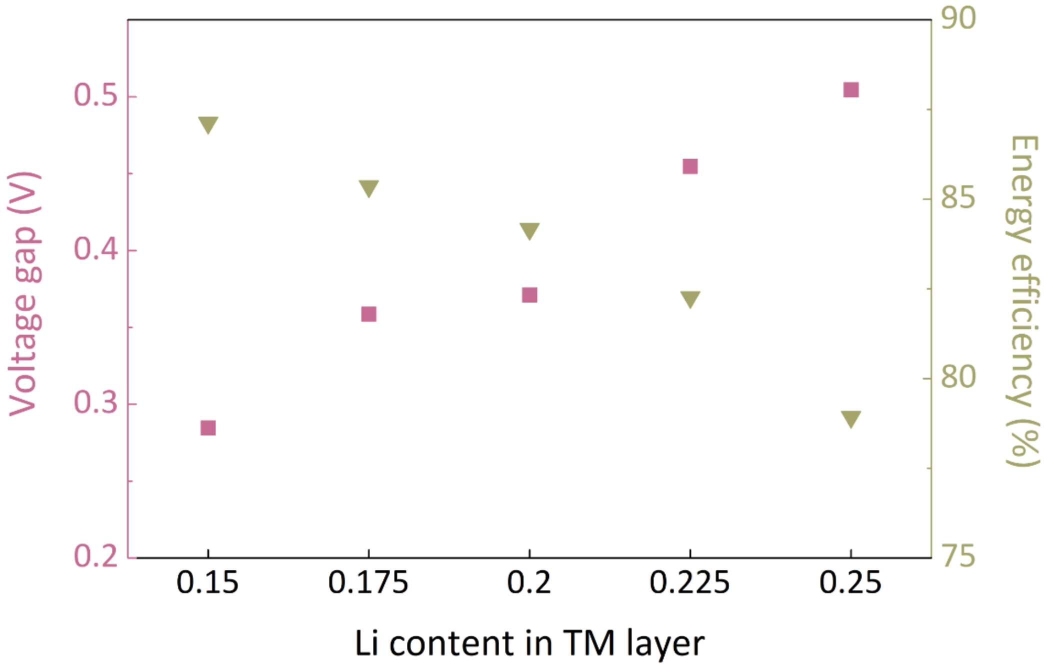

Fig. S13. Average voltage gap between charge and discharge (violet) and energy efficiency (olive) of the second cycles depending on the Li content in TM layer.

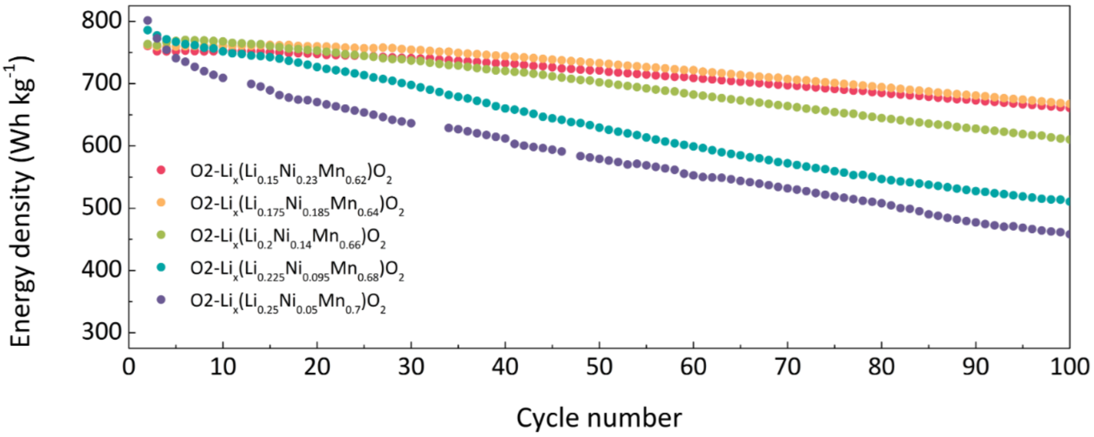

Fig. S14. Specific energy density retention of LL_ys during 100 cycles.

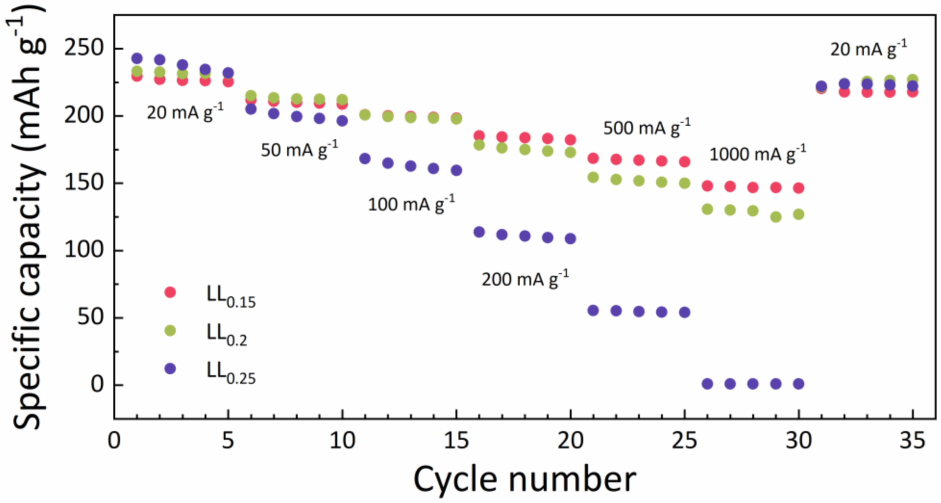

Fig. S15. Rate performances of $LL_{0.15}$,$LL_{0.2}$, and $LL_{0.25}$.

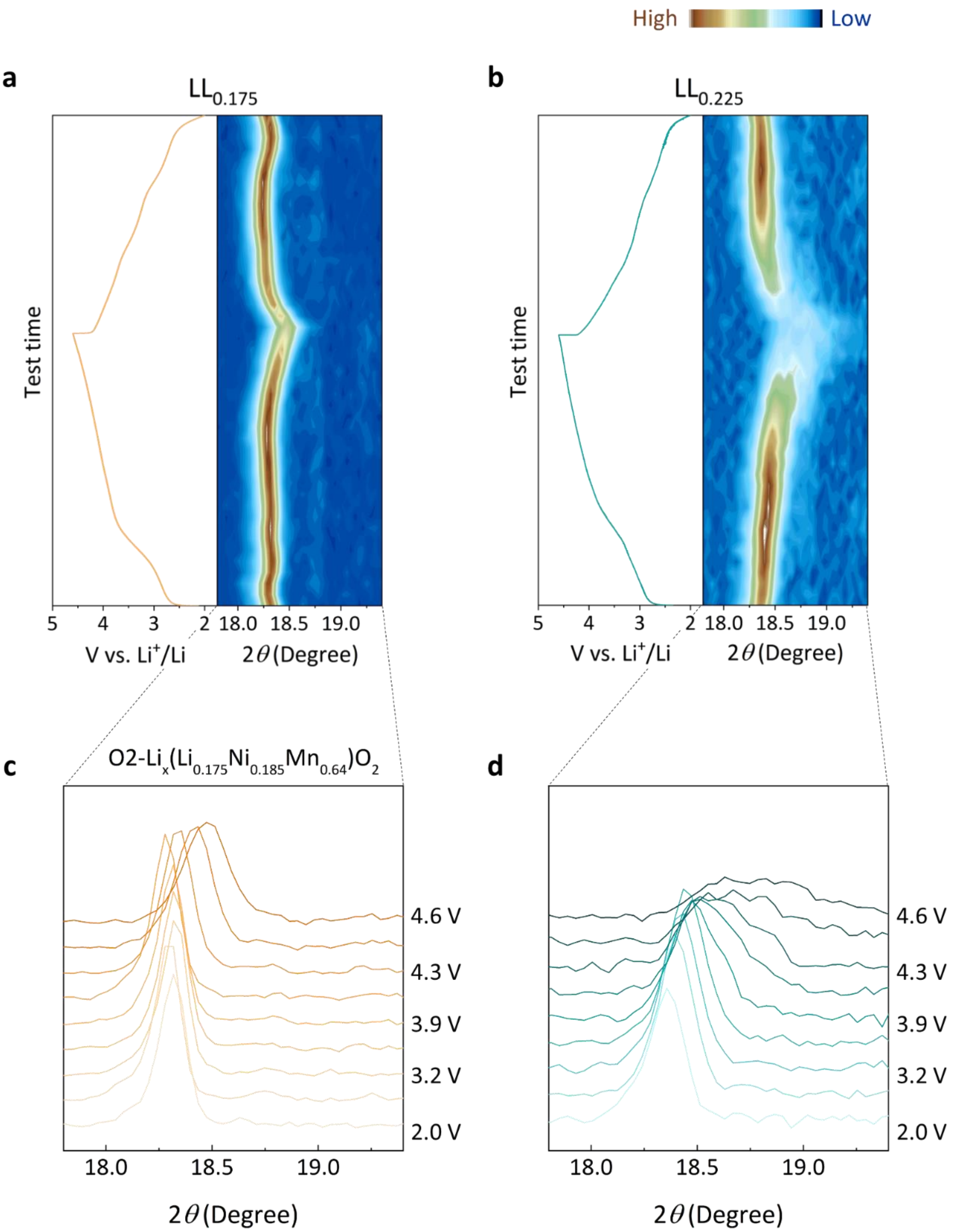

Fig. S16. a,b, Second-cycle charge–discharge profiles and corresponding in situ diffraction patterns (right) for $LL_{0.175}$ (**a**) and $LL_{0.225}$ (**b**). All the cycles were performed within the voltage window of 2.0–4.6 V and at a current density of 20 mA g^{-1}. **c,d,** Magnified (002) XRD peak of $LL_{0.175}$ (**c**) and $LL_{0.225}$ (**d**) during the charging process.

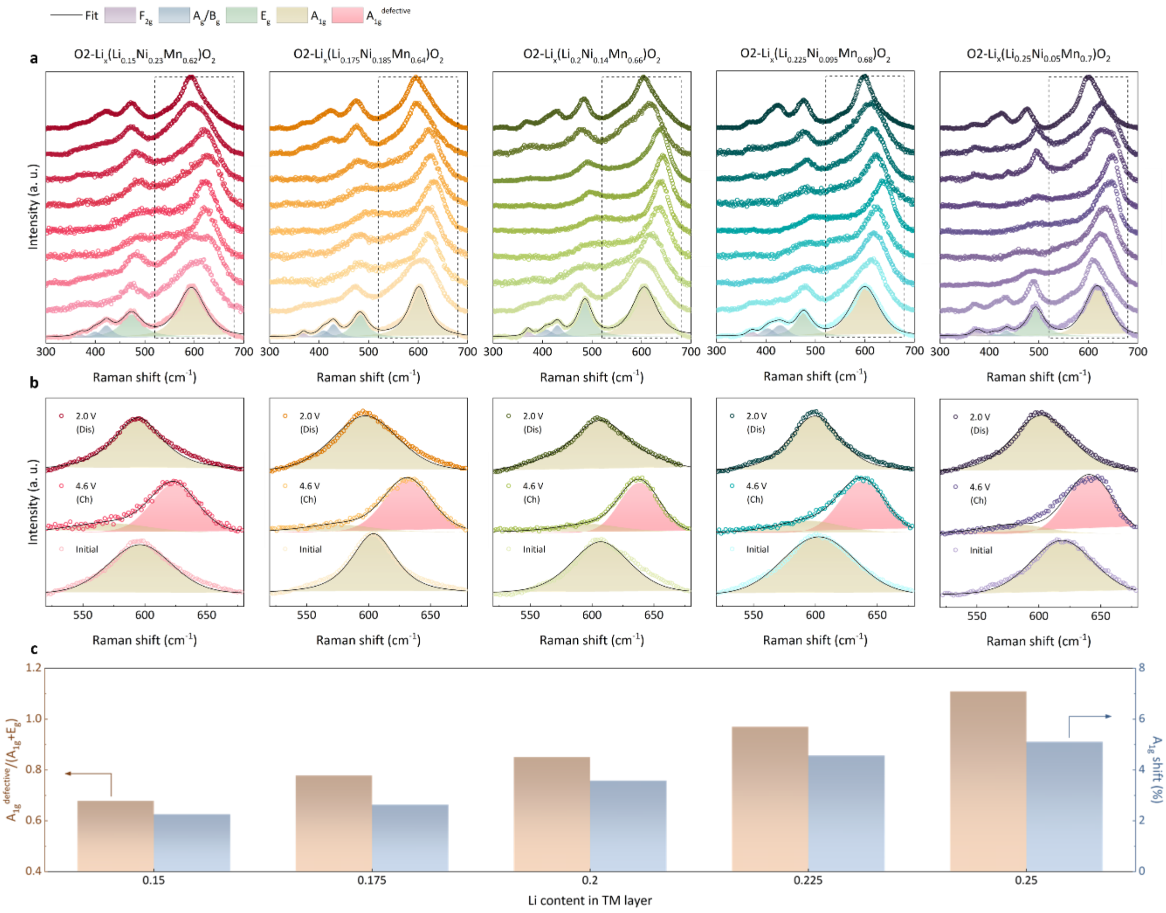

Fig. S17. a, Second-cycle Raman spectra for LL_ys at every quarter of SOCs. **b**, Deconvolution of the peaks in the Raman shift range of 520–680 cm^{-1} of initial (bottom), fully charged (middle), and discharged (top) states for LL_ys. **c**, Comparison of peak-area ratio between the spinel-like peak ($A_{1g}^{defective}$) and intact layered peaks (A_{1g}+E_g) at the end of charge (brown bar) and the degree to which the A_{1g} mode shifted to the left at 4.6 V (blue bar) for LL_ys.

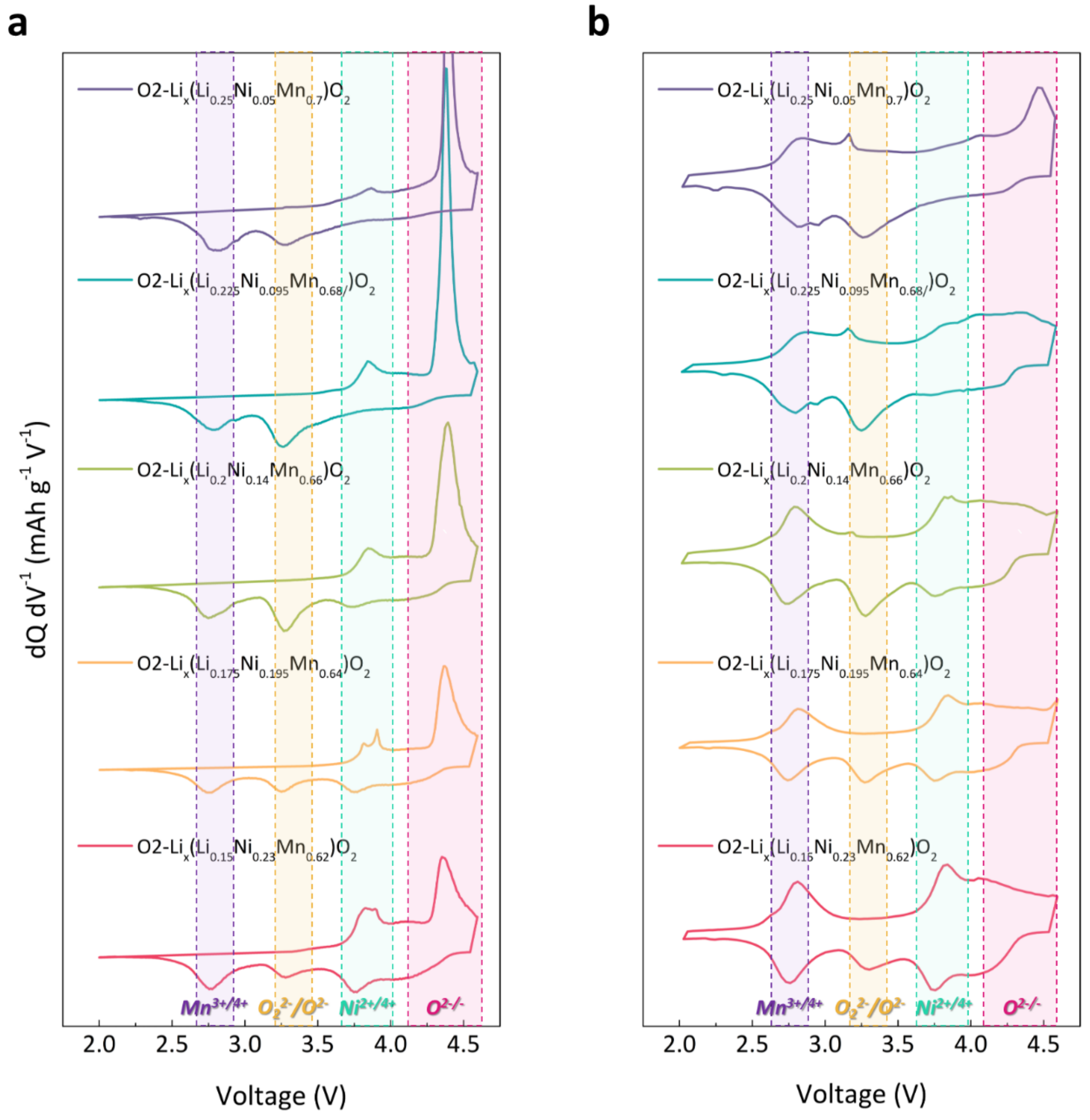

Fig. S18. dQ dV^{-1} plots of $LL_{y}s$ for the charge-discharge curves for the **a**, 1st and **b**, 2nd cycles. The corresponding redox centers were assigned as: $Mn^{3+/4+}$ (purple), polarized O_2^{2-}/O^{2-} (yellow), $Ni^{2+/4+}$ (green), and non-polarized $O^{2-/-}$ (red).

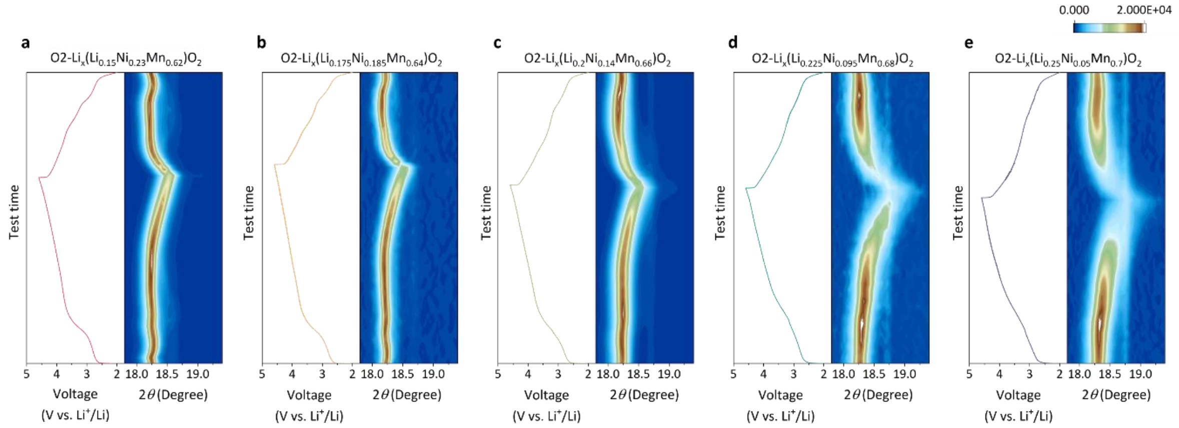

Fig. S19. Third cycle in situ XRD patterns of (002) Bragg peaks for LL_ys (y=0.15 (**a**), 0.175 (**b**), 0.2 (**c**), 0.225 (**d**), and 0.25 (**e**)).

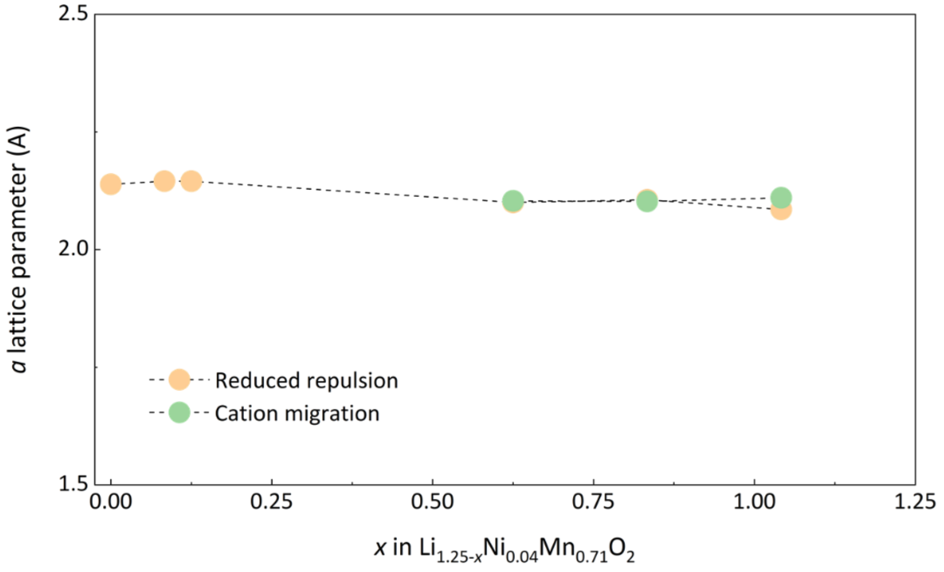

Fig. S20. Theoretical *a*-lattice evolution trend of $LL_{0.25}$ in various delithiation states. It shows far negligible change than *c*-lattice parameter in Fig. 2j.

Li Mn Ni O

Set	Composition		Structure		
	Experimental	Calculation (x ≈ 0.17)	Perfect layered		Migrated
			a-axis	*c*-axis	*a*-axis
$LL_{0.15}$	O2-$Li_x(Li_{0.15}Ni_{0.23}Mn_{0.62})O_2$	O2-$Li_x(Li_{0.17}Ni_{0.21}Mn_{0.62})O_2$	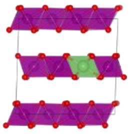	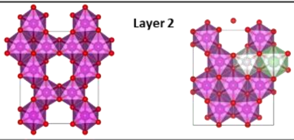	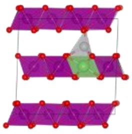
$LL_{0.2}$	O2-$Li_x(Li_{0.2}Ni_{0.14}Mn_{0.66})O_2$	O2-$Li_x(Li_{0.21}Ni_{0.12}Mn_{0.67})O_2$	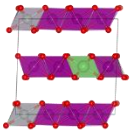	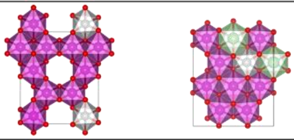	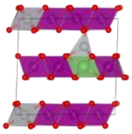
$LL_{0.25}$	O2-$Li_x(Li_{0.25}Ni_{0.05}Mn_{0.7})O_2$	O2-$Li_x(Li_{0.25}Ni_{0.04}Mn_{0.71})O_2$	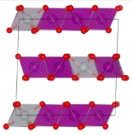	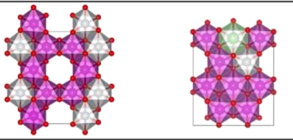	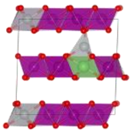

Fig. S21. The nominal compositions of delithiated LL_ys (y=0.15, 0.2, 0.25) in experiments and DFT calculations. The pristine structures are projected from a- and c-direction, while the TM-migrated structure is projected from *a*-direction.

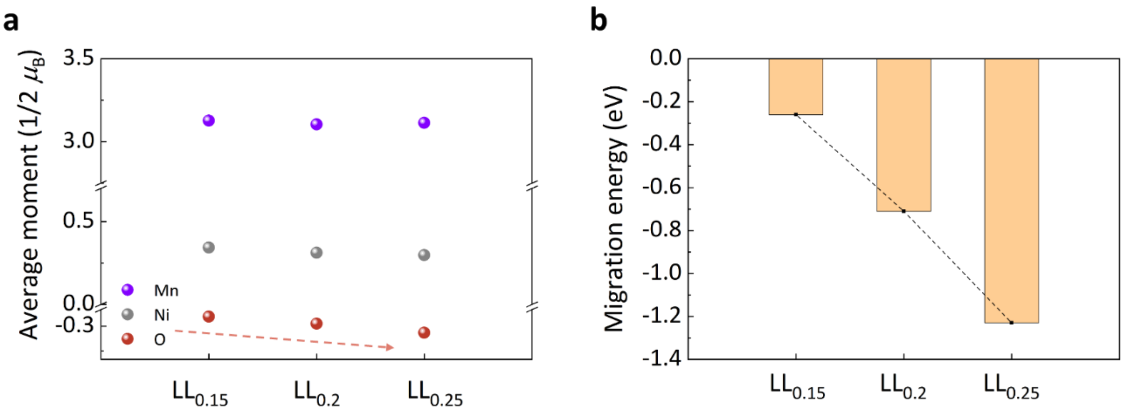

Fig. S22. **a**, Average charge of Ni, Mn and O calculated for the fully charged LL_ys (y=0.15, 0.2, 0.25). Unlike Ni and Mn, in which the charge moments do not change against the compositions, average charge moment of O decreases as the Li content in the material increases, indicating the growing utilization of oxygen redox from $LL_{0.15}$ to $LL_{0.25}$ in line with STXM results in Fig. 1c. **b**, Calculated TM migration energy for the fully charged LL_ys (y=0.15, 0.2, 0.25).

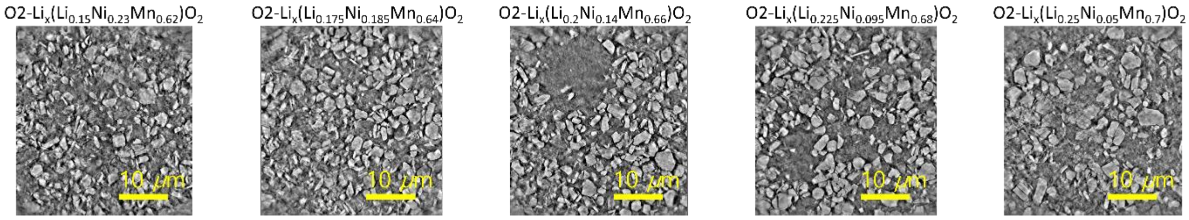

Fig. S23. Cross-sectional images inside the pristine LL_y electrodes from XNI analysis.

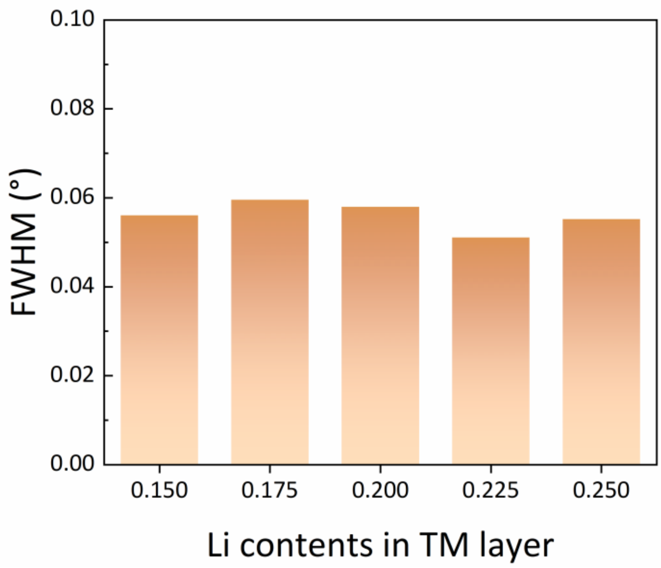

Fig. S24. FWHM values of (002) peaks against the Li contents in TM layer. No distinct correlation between peak broadness and Li contents in TM layer was observed, suggesting comparable bulk stress states among LL_ys.

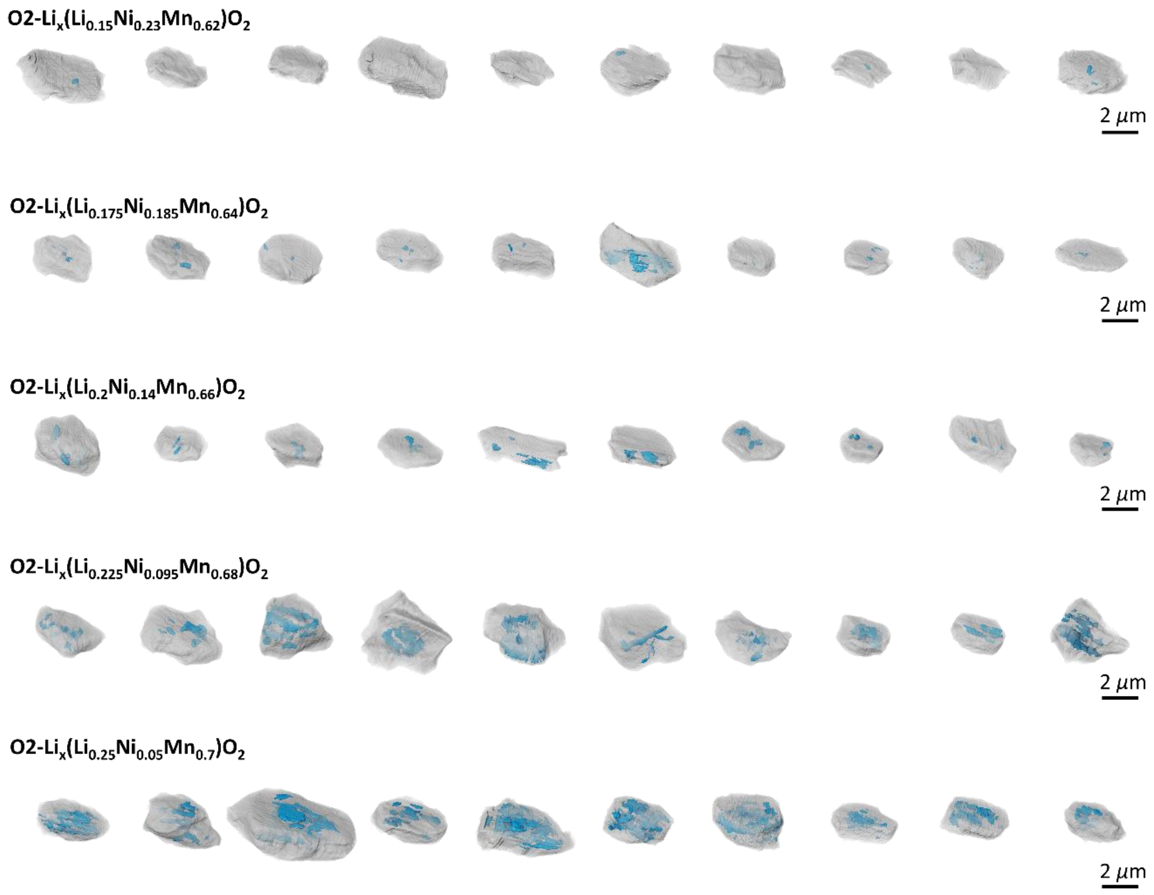

Fig. S25. Ten different 3D-rendered particles for 10th charged LL_ys. The defect area (blue) in the total particle (gray) progressively grows from $LL_{0.15}$ to $LL_{0.25}$.

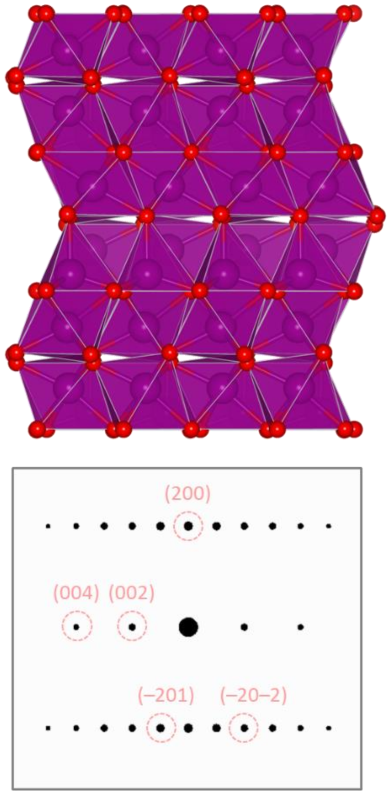

Fig. S26. Simulated SAED pattern along [010] axis for *Pna2₁* disordered Mn_2O_3 phase.

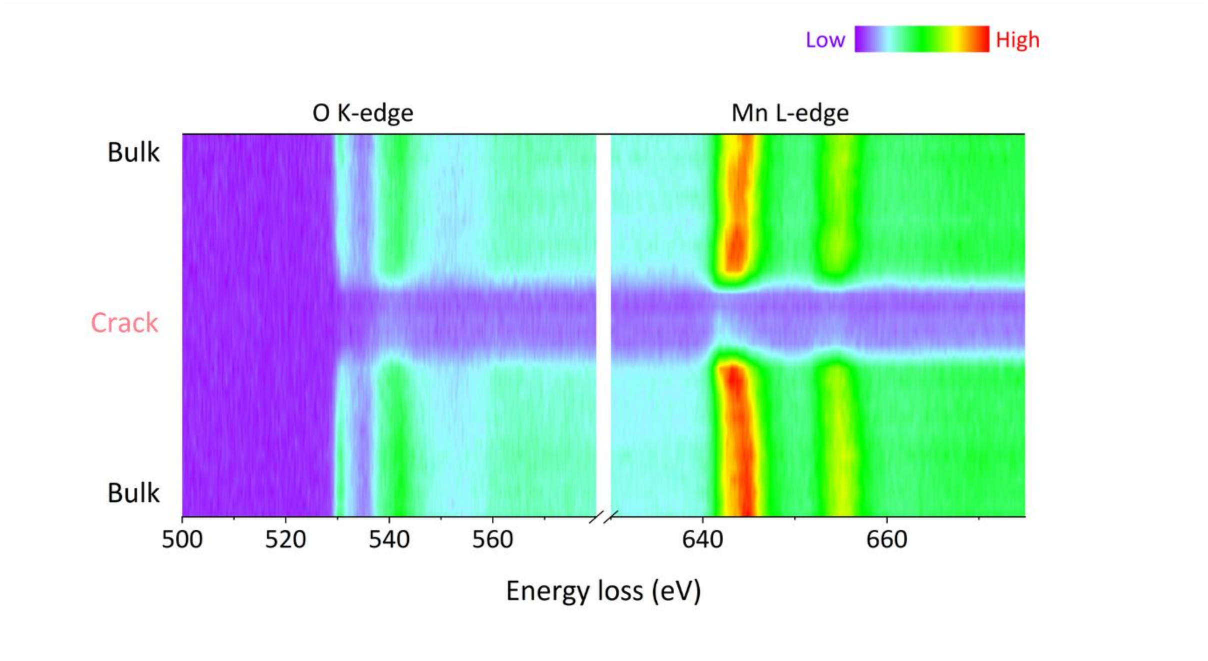

Fig. S27. EELS contour plots for O K- and Mn L-edge across the crack region in Fig. 4g. The color scale indicates arbitrary intensities, increasing from purple to red.

Supplementary Tables

Table S1. ICP-AES results for LL_ys.

	**Na**	**Li**	**Ni**	**Mn**
**$LL_{0.15}$**	0.00262	0.957804	0.236182	0.62
**$LL_{0.175}$**	0.00248	1.007987	0.183807	0.64
**$LL_{0.2}$**	0.0121	1.022116	0.140355	0.66
**$LL_{0.225}$**	0.0051	1.040064	0.093714	0.68
**$LL_{0.25}$**	0.0140	1.075229	0.047757	0.7

Table S2. Relative cation-migration energies of the simulated $LL_{0.15}$, $LL_{0.2}$, and $LL_{0.25}$ electrodes.

**Set**	**Composition**		**Migration energy (eV)**
	Experimental	Calculation (x ≈ 0.17)	
$LL_{0.15}$	O2-$Li_x(Li_{0.15}Ni_{0.23}Mn_{0.62})O2$	O2-$Li_x(Li_{0.17}Ni_{0.21}Mn_{0.62})O2$	–0.26
$LL_{0.2}$	O2-$Li_x(Li_{0.2}Ni_{0.14}Mn_{0.66})O2$	O2-$Li_x(Li_{0.21}Ni_{0.12}Mn_{0.67})O2$	–0.71
$LL_{0.25}$	O2-$Li_x(Li_{0.25}Ni_{0.05}Mn_{0.7})O2$	O2-$Li_x(Li_{0.25}Ni_{0.04}Mn_{0.71})O2$	–1.23

References

1 Myeong, S. *et al.* Understanding voltage decay in lithium-excess layered cathode materials through oxygen-centred structural arrangement. *Nat. Commun.* **9**, 3285 (2018).

2 Gent, W. E. *et al.* Coupling between oxygen redox and cation migration explains unusual electrochemistry in lithium-rich layered oxides. *Nat. Commun.* **8**, 2091 (2017).

3 Hong, J. *et al.* Metal–oxygen decoordination stabilizes anion redox in Li-rich oxides. *Nat. Mater.* **18**, 256-265 (2019).

4 Wu, J. *et al.* Dissociate lattice oxygen redox reactions from capacity and voltage drops of battery electrodes. *Sci. Adv.* **6**, eaaw3871.

5 Ku, K. *et al.* Suppression of Voltage Decay through Manganese Deactivation and Nickel Redox Buffering in High-Energy Layered Lithium-Rich Electrodes. *Adv. Energy Mater.* **8**, 1800606 (2018).

6 Eum, D. *et al.* Voltage decay and redox asymmetry mitigation by reversible cation migration in lithium-rich layered oxide electrodes. *Nat. Mater.* **19**, 419-427 (2020).

7 Eum, D. *et al.* Coupling structural evolution and oxygen-redox electrochemistry in layered transition metal oxides. *Nat. Mater.* **21**, 664-672 (2022).

8 Kim, B. *et al.* A theoretical framework for oxygen redox chemistry for sustainable batteries. *Nat. Sustain.* **5**, 708-716 (2022).

9 Hong, J., Gwon, H., Jung, S.-K., Ku, K. & Kang, K. Review—Lithium-Excess Layered Cathodes for Lithium Rechargeable Batteries. *J. Electrochem. Soc.* **162**, A2447-A2467 (2015).
